# General and specific effects of early-life psychosocial adversities on adolescent grey matter volume^☆^

**DOI:** 10.1016/j.nicl.2014.01.001

**Published:** 2014-01-11

**Authors:** Nicholas D. Walsh, Tim Dalgleish, Michael V. Lombardo, Valerie J. Dunn, Anne-Laura Van Harmelen, Maria Ban, Ian M. Goodyer

**Affiliations:** a Developmental Psychiatry Section, Department of Psychiatry, University of Cambridge, Cambridge, UK; b School of Psychology, Faculty of Social Sciences, University of East Anglia, UK; c Medical Research Council Cognition and Brain Sciences Unit, Cambridge, UK; d Autism Research Centre, Department of Psychiatry, University of Cambridge, Cambridge, UK; e Department of Clinical Neurosciences, University of Cambridge, Addenbrooke's Hospital, Cambridge, UK

**Keywords:** GMV, Grey matter volume, RNLE, recent negative life events, 5-HTTLPR, serotonin-transporter-linked polymorphic region, CA, childhood adversities, ACORN, a classification of residential neighbourhoods, CAMEEI, Cambridge Early Experience Interview, FAD, Family Assessment Device, VBM, voxel based morphometry, PLS, partial least squares, Brain, Life events, Childhood adversity, 5-HTTLPR, Affective disorders, Cerebellum

## Abstract

Exposure to childhood adversities (CA) is associated with subsequent alterations in regional brain grey matter volume (GMV). Prior studies have focused mainly on severe neglect and maltreatment. The aim of this study was to determine in currently healthy adolescents if exposure to more common forms of CA results in reduced GMV. Effects on brain structure were investigated using voxel-based morphometry in a cross-sectional study of youth recruited from a population-based longitudinal cohort. 58 participants (mean age = 18.4) with (n = 27) or without (n = 31) CA exposure measured retrospectively from maternal interview were included in the study. Measures of recent negative life events (RNLE) recorded at 14 and 17 years, current depressive symptoms, gender, participant/parental psychiatric history, current family functioning perception and 5-HTTLPR genotype were covariates in analyses. A multivariate analysis of adversities demonstrated a general association with a widespread distributed neural network consisting of cortical midline, lateral frontal, temporal, limbic, and cerebellar regions. Univariate analyses showed more specific associations between adversity measures and regional GMV: CA specifically demonstrated reduced vermis GMV and past psychiatric history with reduced medial temporal lobe volume. In contrast RNLE aged 14 was associated with increased lateral cerebellar and anterior cingulate GMV. We conclude that exposure to moderate levels of childhood adversities occurring during childhood and early adolescence exerts effects on the developing adolescent brain. Reducing exposure to adverse social environments during early life may optimize typical brain development and reduce subsequent mental health risks in adult life.

## Highlights

• Combined psychosocial factors broadly affect brain grey matter volume (GMV).
• Specific psychosocial risk factors exert specific effects on brain GMV.
• Exposure to childhood adversities reduces medial cerebellar and vermal GMV.
• A subsequent psychiatric history is associated with reduced temporal lobe GMV.
• Exposure to negative life events aged 14 is associated with increased regional GMV.

## 1 Introduction

It is well established that an adverse psychosocial environment in the childhood years significantly increases the risk for later psychopathology (Benjet et al., 2010; Conti et al., 2012; Gilbert et al., 2009; Kessler et al., 2010; Norman et al., 2012; Rutter, 1999; Scott et al., 2012). Psychosocial risk factors can include: low socioeconomic status (SES), poorer parental education, childhood maltreatment, parental psychiatric disorder and exposure to proximal stressful negative life events (Goodyer, 2002; Rutter, 1999). However, there are a number of methodological challenges to consider when examining the effects of exposure to an adverse psychosocial environment upon later behavioral and biological outcome indices. Firstly, these psychosocial risks are inter-correlated creating difficulty in delineating the specific contribution of particular factors in the etiologies of emerging psychopathologies and in their effects upon intermediate neurobiological correlates such as grey matter volume (GMV) (Rutter, 2012a). Secondly, any associations between psychosocial risks and GMV may themselves be a consequence of an ongoing mental illness or may represent residual effects arising from prior psychopathology (Rohde et al., 1994; Wichers et al., 2010). Finally, any effects of experiences occurring in childhood and adolescence occur at a time when the brain is undergoing dramatic structural change (Mills et al., 2014), making it difficult to separate effects of adverse psychosocial experiences from effects due to normative development.

To date the majority of studies investigating main effects of early childhood adversities (CA) on brain structure and function have investigated the impact of exposure to severe forms of physical, sexual or emotional abuse (Hart and Rubia, 2012; McCrory et al., 2012; Northoff, 2013). Currently, five domains of CA have been identified that may adversely impact ongoing mental health: physical abuse, sexual abuse, psychological/emotional abuse, neglect and parental discord with verbal and/or physical violence (Gilbert et al., 2009). Physical or sexual abuse forms of CA are three times less common [UK prevalence rates of around 16% (May-Chahal and Cawson, 2005)] than overt family discord [with prevalence rates of 41% (Dunn et al., 2011)] associated with inter-parental violence and neglect of offspring. Whether the developing brain is sensitive to these more common, family-focused forms of adversities is unclear (Belsky and de Haan, 2011).

Previous structural neuroimaging studies utilizing human and primate models of early-life stress/CA have broadly demonstrated total as well as regional GMV reductions (De Bellis et al., 2002; Sheridan et al., 2012). The main regional reductions have been in the frontal lobe (Dannlowski et al., 2012; De Brito et al., 2013; Hanson et al., 2010), anterior cingulate cortex (ACC) (Baker et al., 2013; Edmiston et al., 2011; Kelly et al., 2013; van Harmelen et al., 2010), amygdala (Hoy et al., 2012; Weniger et al., 2009; Yap et al., 2008), hippocampus (Frodl et al., 2010; Rao et al., 2010; Teicher et al., 2012) and cerebellum (Baldacara et al., 2011a; Bauer et al., 2009; De Bellis and Kuchibhatla, 2006).

The human studies have largely involved scanning young adults with a retrospectively recalled self-report method assessing exposure to prior CA. These associations may however be due to one of more CA-correlated risks that have also shown GMV reductions. For example, current mood/depressive symptoms (Dedovic et al., 2010; Schutter et al., 2012), financial hardship and low SES (Butterworth et al., 2012; Hanson et al., 2011; Noble et al., 2012), parental or familial psychiatric history (Carballedo et al., 2012; Chen et al., 2010; Peterson et al., 2009), previous participant psychiatric history (e.g. MDD, BPD or PTSD) (Carrion et al., 2009; De Bellis et al., 1999; Vythilingam et al., 2002), and recent negative life events (Ansell et al., 2012; Geller et al., 2009; Papagni et al., 2011; Zannas et al., 2013) are all associated with GMV reductions in these regions of interest described above. Studies of early-life stress/CA have however also reported GMV increases or null findings in the amygdala (Landre et al., 2010; Mehta et al., 2009; Tottenham et al., 2010), prefrontal cortex (PFC) (Katz et al., 2009; Richert et al., 2006; Spinelli et al., 2009), ACC (Benedetti et al., 2011; Spinelli et al., 2009), hippocampus (De Bellis et al., 2010; De Brito et al., 2013; Lyons et al., 2001) and cerebellum (Spinelli et al., 2009) regions further complicating our understanding of putative distal CA effects. Additionally there is putative genetic moderation of the liability for a neural effect of CA. Polymorphisms in the 5-HTTLPR genotype have been the most investigated to date with effects reported in the amygdala (Kobiella et al., 2011; Scherk et al., 2009), hippocampal (Everaerd et al., 2012; Frodl et al., 2004), frontal lobe (Jedema et al., 2010; Selvaraj et al., 2011), ACC (Canli et al., 2006; Pezawas et al., 2005), and cerebellar (Canli et al., 2005; Jedema et al., 2010) regions, although negative results have also been reported (Beevers et al., 2010; Cole et al., 2011; Jackowski et al., 2011).

Within this context, it is unclear whether, at the neural systems level, there is a general effect arising from a set of correlated psychosocial risk factors occurring over the childhood and early adolescent years and/or there are specific effects of particular adversities occurring at differing times in the first two decades of life at particular neural regions. In this study we set out to investigate whether adverse psychosocial experiences occurring during the childhood and early adolescent years are associated with variations in GMV in participants now in their later adolescent years. Using a multivariate approach we first tested for a singular general effect of sequentially occurring but related adverse psychosocial variables on later variation in GMV across distributed neural systems. We then tested for associations between specific psychosocial variables and particular neural regions.

## 2 Methods and materials

### 2.1 Recruitment

Participants [N = 58; Mean (SD) age = 18.5 (0.7), range 17–20 years; 35 females] were a subset from the ROOTS study (Total N = 1143), a longitudinal investigation of adolescent emotional development (Goodyer et al., 2010). We generated a list of all potential participants who were eligible based on 5-HTTLPR genotype and prior classification of childhood adversity (CA) (see below). The selection and recruitment process is described in more detail in Walsh et al. (2012). Participants recruited to the neuroimaging sub-study showed no significant selection bias compared to the total ROOTS sample in terms of gender ratio or socioeconomic status as assessed using the ACORN (A Classification Of Residential Neighbourhoods) geodemographic measure (Morgan and Chinn, 1983) (http://www.caci.co.uk). As a brief background, the ACORN classification is built entirely using Census data and includes information on age, sex, marital status occupation, economic position, education, home ownership and car ownership. However, participants in the neuroimaging study had lower levels of self-reported depressive symptoms at the time of scanning relative to the overall ROOTS sample (measured age 17).

The study was carried out in accordance with the Declaration of Helsinki and Good Clinical Practice guidelines and approved by the Cambridgeshire Research Ethics Committee. All participants provided written informed consent. Inclusion criteria for the neuroimaging sub-study were as follows: normal or corrected-to-normal vision; English speaking; and of Northern European descent (to facilitate genetic allele comparisons). Exclusion criteria were: any history of neurological trauma resulting in loss of consciousness; current psychotropic medication use; current neurological disorder; current DSM-IV Axis 1 disorder; presence of metal in body; specific learning disability, and IQ < 85 on the Weschler Abbreviated Scale of Intelligence (Wechsler, 1999).

### 2.2 Assessment of childhood adversities (0–11 years) — the Cambridge Early Experiences Interview (CAMEEI)

This semi-structured interview is conducted with the child's primary caregiver and records family-focused adverse life experiences, child's age at occurrence, duration, and an interviewer assessment of their practical impact on the daily life of the family (see (Dunn et al., 2011) for more information). The current investigation used information covering the first eleven years of life to classify adolescents into those exposed (CA +, n = 27) and not exposed (CA −, n = 31) to early CA. The first eleven years was covered in order to make our groups comparable to the age limit for early maltreatment used in the maltreatment studies by Caspi and colleagues (Caspi et al., 2002, 2003). Exposure to an adverse family environment was defined as exposure to abuse (emotional, physical or sexual) and/or significant family discord; occasional physical violence, lack of affectionate warmth, or severe lack of communication between family members. In summary, amongst the 27 CA + participants, none had sexual abuse. For physical abuse, 2 (7%) were classified as possibly being exposed, 1 (4%) was classified as yes/probably being exposed. For emotional abuse 4 (15%) were classified as yes/probably being exposed. All 27 had been exposed to moderate to severe inter-parental discord. Exposure was estimated to begin from birth with the duration estimated as ranging from 5 through to 56 months (mean 30.8 (sd 26.1) months).

### 2.3 Description of participant psychiatric history

Participants were longitudinally assessed for a past psychiatric diagnosis through their participation in the ROOTS study (using the Kiddie Schedule for Affective disorders and Schizophrenia for School-Age Children (Kaufman et al., 1997) assessments). Retrospective inspection of the ROOTS data-set revealed that 18 participants (31%) had prior DSM-IV diagnoses and these are reported in Inline Supplementary Table S1.

Participants were longitudinally assessed for a past psychiatric diagnosis through their participation in the ROOTS study (using the Kiddie Schedule for Affective disorders and Schizophrenia for School-Age Children (Kaufman et al., 1997) assessments). Retrospective inspection of the ROOTS data-set revealed that 18 participants (31%) had prior DSM-IV diagnoses and these are reported in Inline Supplementary Table S1.

Inline Supplementary Table S1

**Table S1:** Neuroimaging sample participant psychiatric history as diagnosed using the K-SADS.

Group	Previous disorder
CA +	Previous NSSI, affective disorder(MDD), anxiety disorder (Panic disorder)
CA +	Previous anxiety disorder (Specific phobia)
CA +	Previous anxiety disorder (Specific phobia)
CA +	Previous NSSI, affective disorder (MDD), Anxiety disorder (Anxiety NOS)
CA +	Previous NSSI
CA +	Previous behavioral disorder (CD, ODD, ADHD)
CA +	Previous anxiety disorder (Panic disorder)
CA-	Previous affective disorder (MDD), anxiety disorder (Specific spider phobia)
CA-	Previous affective disorder (MDD), previous anxiety disorder (Panic attack)
CA-	Previous NSSI, previous MDD
CA-	Previous anxiety disorder (OCD & Panic attacks)
CA +	Previous NSSI, affective disorder (MDD), anxiety disorder (Panic disorder), alcohol abuse
CA +	Previous NSSI, affective disorder MDD, anxiety disorder (Panic disorder)
CA +	Previous NSSI
CA +	Previous eating disorder
CA +	Previous affective disorder (MDD)
CA-	Previous behavioral disorder (ADHD)
CA-r	Previous alcohol abuse

**Abbreviations:** NSSI (Non-Suicidal Self Injury). MDD (Major Depressive Disorder). NOS (Not Otherwise Specified). CD (Conduct Disorder). ODD (Oppositional Defiant Disorder). OCD (Obsessive Compulsive Disorder). ADHD (Attentional Deficit Hyperactivity Disorder).

Inline Supplementary Table S1

Inline Supplementary Table S1 can be found online at http://dx.doi.org/10.1016/j.nicl.2014.01.001.

### 2.4 Assessment of parental psychiatric history

The MINI Mental State Examination (Sheehan et al., 1998) was embedded within the CAMEEI assessment to assess parental mental illness during the participant's childhood. We also recorded disorder in biological parents prior to the birth of the participant and when living away from the family. Thresholds for inclusion were set very high with clear evidence of impairment essential for diagnosis. As most interviewees were mothers, the information on maternal mental health is the most reliable and valid and was corroborated using clinical notes. Parental psychiatric diagnoses are reported in Inline Supplementary Table S2.

The MINI Mental State Examination (Sheehan et al., 1998) was embedded within the CAMEEI assessment to assess parental mental illness during the participant's childhood. We also recorded disorder in biological parents prior to the birth of the participant and when living away from the family. Thresholds for inclusion were set very high with clear evidence of impairment essential for diagnosis. As most interviewees were mothers, the information on maternal mental health is the most reliable and valid and was corroborated using clinical notes. Parental psychiatric diagnoses are reported in Inline Supplementary Table S2.

Inline Supplementary Table S2

**Table S2:** Neuroimaging sample - parental psychiatric history.

Group	Parental diagnosis
CA +	Mother Anxiety Disorder/MDD
CA +	Mother MDD
CA +	Mother MDD
CA-	Father MDD
CA-	Father MDD
CA +	Mother MDD
CA-	Mother MDD
CA +	Mother MDD, Father MDD
CA +	Mother MDD, Father MDD
CA-	Father MDD
CA-	Mother MDD
CA-	Mother MDD
CA +	Mother MDD
CA +	Mother Dysthymia
CA +	Mother MDD
CA +	Father Alcohol/Substance Abuse
CA +	Mother MDD, Father Alcohol/Substance Abuse
CA +	Mother Eating Disorder, MDD
CA +	Mother MDD
CA +	Father MDD
CA +	Mother MDD, Father Alcohol/Substance Abuse
CA-	Mother Anxiety Disorder
CA-	Father MDD
CA-	Mother MDD, Father Bipolar Disorder
CA +	Mother MDD, Personality Disorder
CA-	Mother MDD
CA-	Mother MDD
CA +	Mother MDD
CA +	Mother MDD and Dysthymia
CA +	Mother MDD
CA +	Mother MDD
CA +	Mother Dysthymia, Father Alcohol/Substance Abuse
CA +	Mother MDD

**Abbreviations:** MDD (Major Depressive Disorder)

Inline Supplementary Table S2

Inline Supplementary Table S2 can be found online at http://dx.doi.org/10.1016/j.nicl.2014.01.001.

### 2.5 Genotyping for 5-HTTLPR

DNA was harvested from separate saliva samples (Qiagen, Crawley, UK) and genotyped for 5-HTTLPR. The 5-HTTLPR region was amplified using the primers 5-ATGCCAGCACCTAACCCCTAATGT-3 and 5-GGACCGCAAGGTGGGCGGGA-3, which generates a 419 bp and 375 bp product for the “l” and “s” alleles respectively. The PCR reaction mixture consisted of: 100 ng genomic DNA, 10 mM Tris–HCl (pH 9.0), 1.5 mM MgCl_2_, 50 mM KCl, 0.1% Triton® X-100, 1.25 U *Taq* DNA polymerase, 200 μM dNTPs, 500 nM each of forward and reverse primer and 100 μM 7-Deaza-dGTP in a final reaction volume of 15 μL. The reaction conditions were 98 °C for 7 min, followed by 40 cycles of 96 °C for 30 s, 61 °C for 30 s and 72 °C for 1 min with a final extension stage of 72 °C for 10 min. PCR products were electrophoresed on a 3700 DNA analyser (Applied Biosystems) with semi-automated sizing and genotyping performed using GENESCAN v3.7 and GENOTYPER v3.7 software (Applied Biosystems). The 5-HTTLPR frequency in the ROOTS cohort as a whole was in Hardy–Weinberg equilibrium (LL = n = 352; 30.3%; LS = n = 596; 51.3%; SS = n = 214; 18.4%), *x*2 = 3.99, df = 2, p = .14. The neuroimaging sub-study was confined to participants with either the l/l or s/s genotype in order to maximize potential statistical differences between alleles, as conducted in prior experimental neurogenetic studies (Firk et al., 2013). Additionally, meta-analyses have often demonstrated differences between l/l and s/s homozygotes on outcome variables [e.g. cortisol reactivity (Miller et al., 2013), association with anxiety traits (Minelli et al., 2011), and hypertension (Zhang et al., 2013)] but not differences between heterozygotes (s/l participants) and l/l participants. We also performed a secondary analysis classifying participants according to the rs25531 SNP. For this analysis we collapsed the low-expressing SS and LaLg variant participants (n = 32) and compared against participants with the high-expressing LaLa variants (n = 26) as performed in prior studies e.g. Hu et al. (2006) and Praschak-Rieder et al. (2007).

### 2.6 Assessment of recent negative life events (RNLE) aged 13–14 & 16–17

At ages 14 and 17, participants in the ROOTS cohort had completed a self-report measure of negative life events and difficulties [modified from Goodyer et al. (2000)], occurring to them, their family or closest friends over the preceding 12 months. Participants were asked to date these experiences and rate their impact on themselves on a scale from 1 = very pleasant/happy to 5 = very unpleasant/sad/painful. If participants rated either 4 or 5 they were asked to indicate if they felt upset for longer than 2 weeks. From these ratings, two separate summed totals for positive and negative recent life events rated as occurring for longer than 2 weeks were derived. The negative event ratings were used here.

### 2.7 Family Assessment Device — Global Functioning Subscale (FAD-GF)

The FAD-GF (Epstein et al., 1983; Miller et al., 2000) is a 12-item self-report scale measuring overall health/pathology of the family. Six items describe healthy functioning and the other six describe unhealthy functioning. Each item is rated on a 4 point Likert scale (4 = ‘strongly agree’, 3 = ‘agree’, 2 = ‘disagree’, 1 = ‘strongly disagree’). The higher the score the worse the family functioning.

### 2.8 Assessment of current depressive symptoms at time of scanning

The Mood and Feelings Questionnaire (MFQ) (Angold et al., 1995) was used to assess current depressive symptoms in the two weeks prior to scanning. This assessment was administered on the day of the scanning session.

### 2.9 Image acquisition and preprocessing

Structural MRI data were acquired using a 3-T Siemens Tim Trio scanner at the MRC Cognition and Brain Sciences Unit, Cambridge, UK. We acquired T1-weighted 3D magnetization-prepared rapid acquisition with gradient-echo images (voxel size = 1 × 1 × 1 mm, repetition time = 2250 ms, echo time = 2.99 ms, inversion time = 900 ms, flip angle = 9°). Total scanning time was 4 min 16 s.

Preprocessing for voxel-based morphometry (VBM) was performed using SPM8 software (Welcome Trust Center for Neuroimaging, London, UK, http://www.fil.ion.ucl.ac.uk/spm/software/spm8/). Data was first checked visually for scanner artifacts and gross anatomical abnormalities for each subject and the origin of all images was aligned with the anterior commissure. Next, initial segmentation of images into grey-matter (GM) and white-matter (WM) was implemented using the ‘New Segment’ option in the SPM8 DARTEL toolbox (Ashburner, 2007). These native space GM and WM images were then aligned in an iterative fashion using the high-dimensional non-linear diffeomorphic registration algorithm employed by DARTEL in order to create a study-specific template (Ashburner, 2007). Non-linear warping parameters (i.e. flow fields) estimated from the template creation step were then used to spatially normalize and modulate the data to standard space (Montreal Neurological Institute; MNI) in order to preserve local volumetric information at the voxel-wise level. Finally, normalized modulated maps were smoothed using a 4 mm FWHM kernel. This kernel was chosen to optimize further analyses to be sensitive to very small localized differences in subcortical structures (e.g. amygdala). Furthermore because DARTEL achieves much more accurate registration than previous warping techniques (Klein et al., 2009), registration error is reduced and requires less smoothing for correction.

### 2.10 Data analytic strategy

#### 2.10.1 Association of psychosocial variables in ROOTS and neuroimaging subsample

In order to show that the neuroimaging sample was representative of the larger population-level ROOTS cohort, we compared the association of psychosocial variables in the ROOTS cohort and neuroimaging sub-sample (CA, RNLE14, RNLE17, previous psychiatric history, parental psychiatric history, FAD score, MFQ score). Mann–Whitney and Phi coefficient tests were run on the categorical and continuous level variables described above.

#### 2.10.2 Multivariate associations between adverse psychosocial experience and brain GMV

In our first analysis we investigated whether the combination of adverse psychosocial variables identified previously (CA, RNLE14, RNLE17, previous psychiatric history, parental psychiatric history, FAD score, MFQ score), was associated with whole-brain GMV. Here we used partial-least squares (PLS) (McIntosh et al., 1996) analysis implemented using the PLSGUI software (http://www.rotman-baycrest.on.ca/pls/) (Krishnan et al., 2011; McIntosh and Lobaugh, 2004; McIntosh and Misic, 2013). A permutation test (1000 permutations) evaluated the significance of latent brain–behavior pairs and 1000 bootstrap resamples were used to assess the reliability of voxels with the strongest contribution to the pattern. For visualization of the most reliable voxels contributing to the patterns, we used a bootstrap ratio of 3 and an extent threshold of 250 voxels. The bootstrap ratio can be viewed/interpreted as a pseudo Z-statistic, since it is the ratio of a voxel's ‘salience’ (i.e. a latent variable linear combination of the original variables) divided by the standard error estimated from bootstrapping (McIntosh and Lobaugh, 2004). This bootstrap ratio allows us to infer which voxels were most important and reliable to contributing to the overall pattern picked up by PLS.

#### 2.10.3 Specific psychosocial variable associations on regional GMV

We performed follow-up univariate analyses in SPM8. A 2-sample *t*-test was run with CA as the dependent variable and the following variables as covariates: participant and parental psychiatric history, RNLE14, RNLE17, current depressed mood, FAD score, 5-HTTLPR genotype, gender and total-intracranial volume (TIV). In Section 3.3 we first report the CA effect and then any significant covariate effects. We performed a whole-brain analysis in SPM and subsequently discuss only regions surviving either stringent Family-Wise Error (FWE) correction at p < 0.05 (Worsley et al., 1996) or using cluster-False Discovery Rate correction for multiple comparisons at q < 0.05 (Chumbley et al., 2010) while using non-stationarity of smoothness correction (Hayasaka et al., 2004).

#### 2.10.4 Commonality between the multivariate and univariate analyses

To demonstrate that the regions associated with CA + (identified in the univariate analysis) were the same regions identified in the multivariate analysis, we used a logical AND masking procedure on the whole-brain-corrected results maps to implement conjunction analyses (Nichols et al., 2005).

## 3 Results

### 3.1 Participant characteristics

In Table 1 we report participant characteristics of the neuroimaging subsample classified according to CA grouping. In the ROOTS total sample there were no significant differences of age, gender or 5-HTTLPR genotype ratio on CA. However, the presence of childhood adversity (CA +) in the total sample was associated with significantly lower familial SES (*p* < 0.001, *r* = − 0.18), more lifetime diagnoses of psychiatric disorder (*p* < 0.001, *r* = − 0.16), increased parental psychiatric disorder (*p* < 0.001, *r* = − 0.31), more negative current perceptions of family functioning (*p* < 0.001, *r* = − 0.17), increased depressive symptoms at age 17 (*p* < 0.001, *r* = − 0.13), and increased reporting of negative life events at age 14 (*p* < 0.05, *r* = − 0.06).

In the neuroimaging sub-sample, the presence of childhood adversities (CA +) was associated with significantly more lifetime diagnoses of psychiatric disorder (*p* < 0.05, *r* = − 0.27), significantly higher parental psychiatric disorder (*p* < 0.01, *r* = − 0.27), and significantly higher negative current perceptions of family functioning, as assessed with the FAD-GF (*p* < 0.05, *r* = − 0.31) relative to the CA − groups.

### 3.2 Multivariate structural imaging analysis

**Fig. 1:**
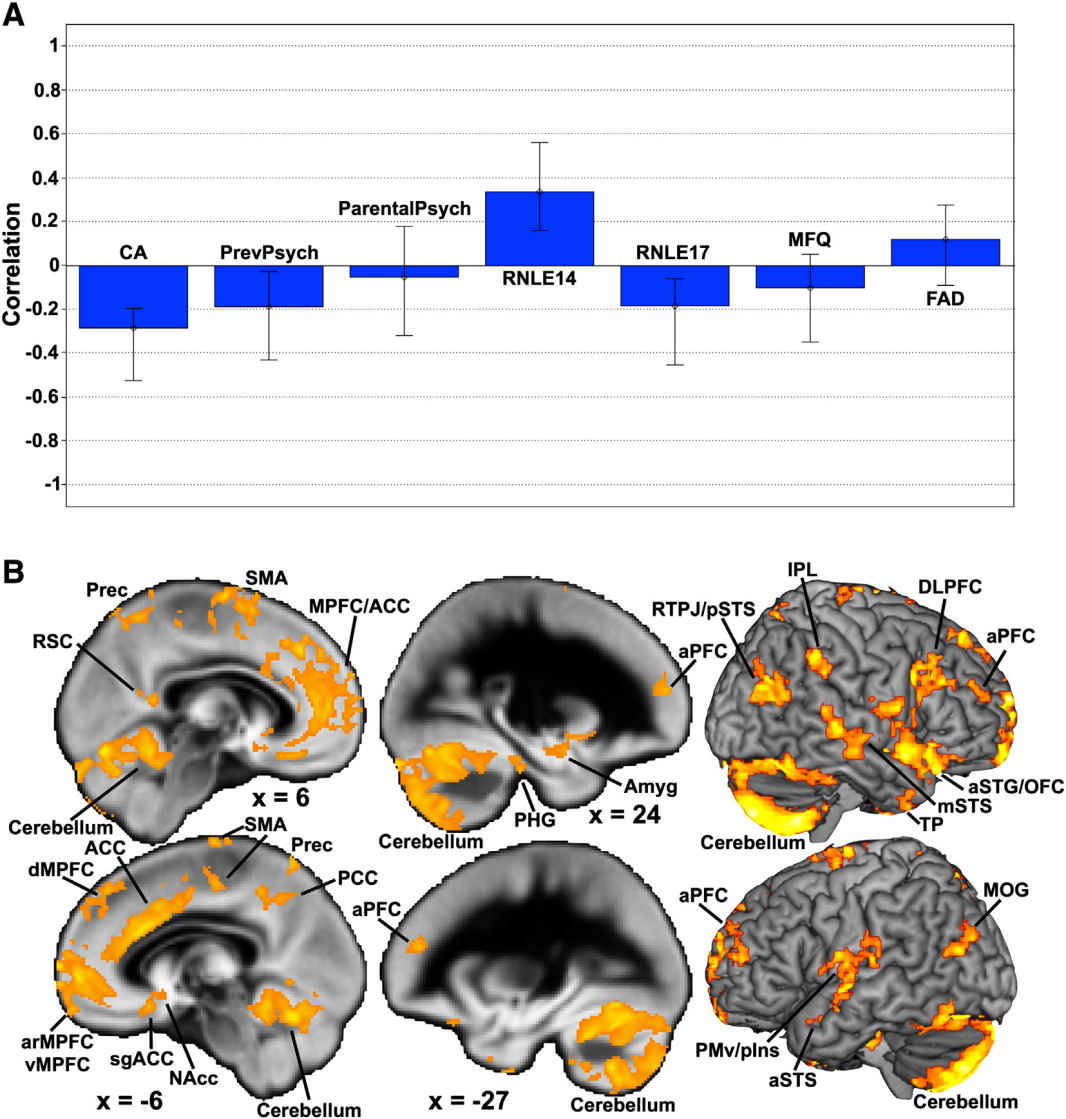
PLS results. Panel A shows the PLS behavioural saliences transformed into correlations that depict each psychosocial variable's contribution and directionality to the overall multivariate effect of influence on GMV. Error bars are the 95% confidence intervals estimated from bootstrapping. Panel B shows the most reliable brain regions that contribute to the latent brain–behavior pair identified by PLS. Abbreviations: CA, childhood adversity; PrevPsych; previous psychiatric history; ParentalPsych, parental psychiatric history; RNLE14, recent negative life events at 14 years old; RNLE17 recent negative life events at 17 years old; MFQ, Mood and Feelings Questionnaire; FAD, Family Assessment Device; RSC, retrosplenial cortex; Prec, precuneus; SMA, supplementary motor area; MPFC, medial prefrontal cortex; ACC, anterior cingulate cortex; dMPFC, dorsomedial prefrontal cortex; arMPFC, anterior rostral medial prefrontal cortex; vMPFC, ventromedial prefrontal cortex; sgACC, subgenual anterior cingulate cortex; NAcc, nucleus accumbens; PCC, posterior cingulate cortex; aPFC, anterior prefrontal cortex; PHG, parahippocampal gyrus; Amyg, amygdala; RTPJ, right temporo-parietal junction; pSTS, posterior superior temporal sulcus; IPL, inferior parietal lobule; DLPFC, dorsolateral prefrontal cortex; aSTG, anterior superior temporal gyrus; OFC, orbitofrontal cortex; mSTS, mid superior temporal sulcus; TP, temporal pole; MOG, middle occipital gyrus; aSTS, anterior superior temporal sulcus; PMv, ventral premotor cortex; vIns, ventral insula.

PLS identified only one significant latent brain–behavior pair which accounted for 47.70% of the covariance between GMV and adverse psychosocial variables (d = 179.16, permutation p < 0.001). Fig. 1A shows the PLS behavioral saliences (transformed into correlations for ease of interpretation) and the error bars show the 95% confidence intervals estimated from bootstrapping. This shows negative associations between a cluster of psychosocial variables (CA and previous psychiatric history, increased RNLE17 exposure and higher depression scores) and GMV across multiple brain regions. There was also one positive association between RNLE14 and GMV.

The brain regions where this pattern was most reliably identified can be seen in Fig. 1B. The affected brain regions are widely distributed and encompass the cerebellum, anterior, medial, lateral and orbital PFC, ACC, subgenual ACC, medial parietal regions, amygdala, nucleus accumbens, superior temporal gyrus/sulcus and temporal pole (see Fig. 1B and Table 2).

### 3.3 Univariate structural imaging analyses

#### 3.3.1 Childhood adversities and cerebellum

In the first analysis, we tested for regions demonstrating reduced GMV in individuals exposed to CA (CA +) compared to non-exposed individuals (CA −). This analysis was run whilst covarying for potential confounds described above.

**Fig. 2:**
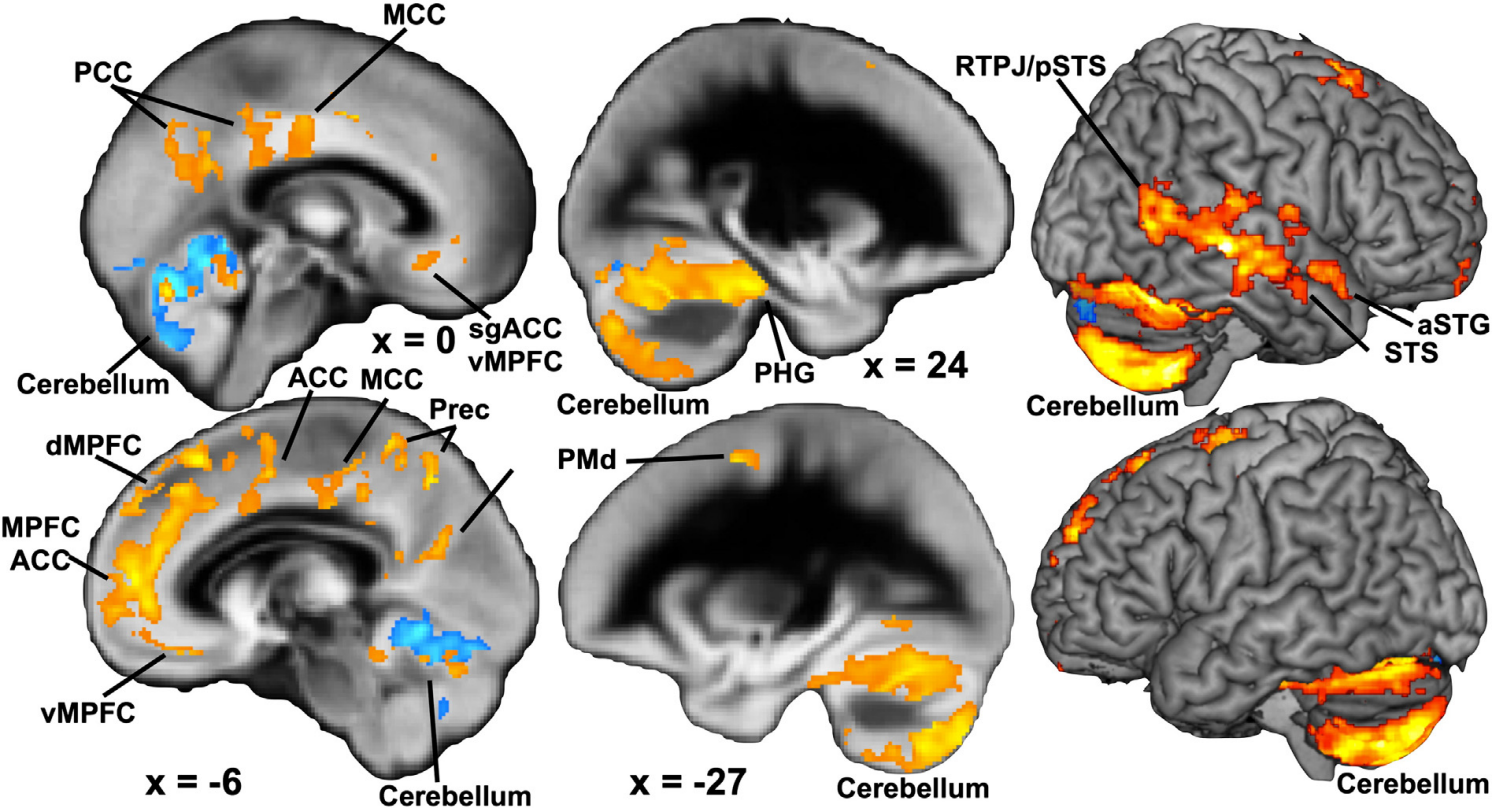
Univariate results of CA and RNLE14 effects. This figure shows brain regions where the presence of childhood adversities (CA) was related to decreased GMV (blue voxels) or where increased recent negative life events at 14 years of age (RNLE14) was related to increased GMV (orange voxels). These effects were found after partialling out variability due to other psychosocial variables and are whole-brain corrected at a cluster-FDR of q < 0.05. Abbreviations: MCC, middle cingulate cortex; PCC, posterior cingulate cortex; Prec, precuneus; dMPFC, dorsomedial prefrontal cortex; MPFC, medial prefrontal cortex; ACC, anterior cingulate cortex; sgACC, subgenual anterior cingulate cortex; vMPFC, ventromedial prefrontal cortex; PMd, dorsal premotor cortex; PHG, parahippocampal gyrus; RTPJ, right temporo-parietal junction; pSTS, posterior superior temporal sulcus; STS, superior temporal sulcus; aSTG, anterior superior temporal gyrus.

Whole-brain analysis showed that individuals exposed to CA (CA +) compared to CA − individuals demonstrated significantly reduced GMV mainly in medial cerebellar lobes V and VI and vermis regions Crus II, VI, VIIb and VIIIa (see blue voxels in Fig. 2 and Table 3). There were no regions demonstrating greater GMV in individuals exposed to CA compared to non-exposed individuals.

Reduced cerebellar GMV was not associated with age of CA onset, duration or severity of exposure.

#### 3.3.2 Participant psychiatric history and medial temporal lobe

**Fig. 3:**
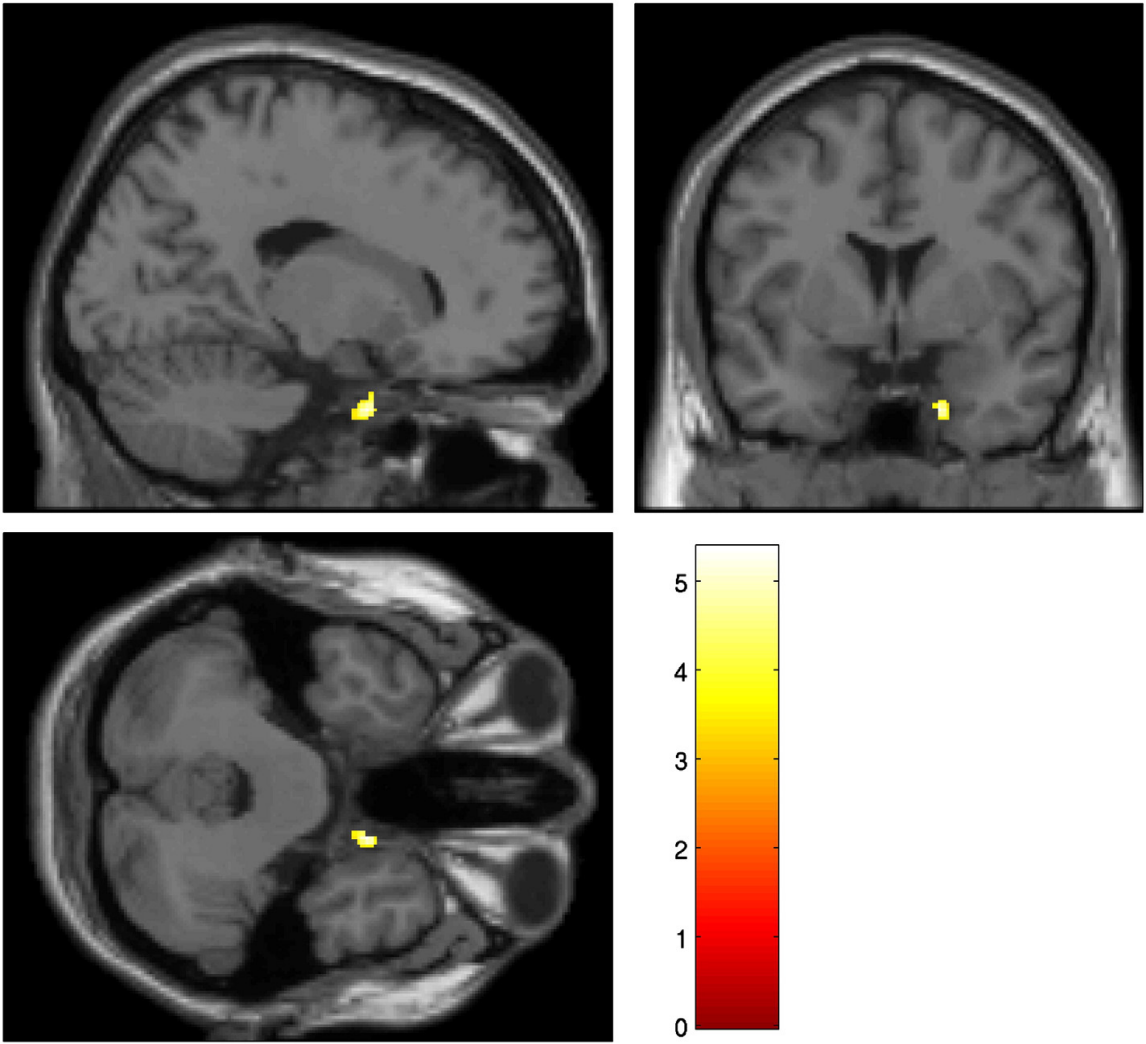
Whole-brain univariate results of participant psychiatric history. This figure shows the right uncus/parahippocampal region whereby participants reporting a psychiatric history demonstrated decreased GMV compared to non-reporting participants. These effects were found after partialling out variability due to other psychosocial variables. Activation thresholded at p < 0.001.

Participants with a lifetime history of psychiatric illness (see Inline Supplementary Table S1) demonstrated reduced GMV in the right uncus/parahippocampal region (p = 0.042 FWE, whole-brain correction) (see Table 3 and Fig. 3).

Participants with a lifetime history of psychiatric illness (see Inline Supplementary Table S1) demonstrated reduced GMV in the right uncus/parahippocampal region (p = 0.042 FWE, whole-brain correction) (see Table 3 and Fig. 3).

#### 3.3.3 Recent negative life events aged 14 and cerebellar, cingulate and cortical structures

Whole-brain analysis showed an association between recent negative life events recalled at 14 years of age for the previous 12 months and increased GMV within cortical midline regions such as medial prefrontal cortex, anterior, mid and posterior cingulate cortices, and precuneus, as well as lateral cerebellar regions and right superior temporal gyrus (see Table 3).

#### 3.3.4 Other modeled covariates

There were no significant effects of parental psychiatric history, current depressive symptoms, FAD score, RNLE17, or 5-HTTLPR genotype (with the original biallelic or rs25531 classifications) upon adolescent GMV at the whole-brain level.

### 3.4 Overlap between multivariate and univariate analyses

**Fig. 4:**
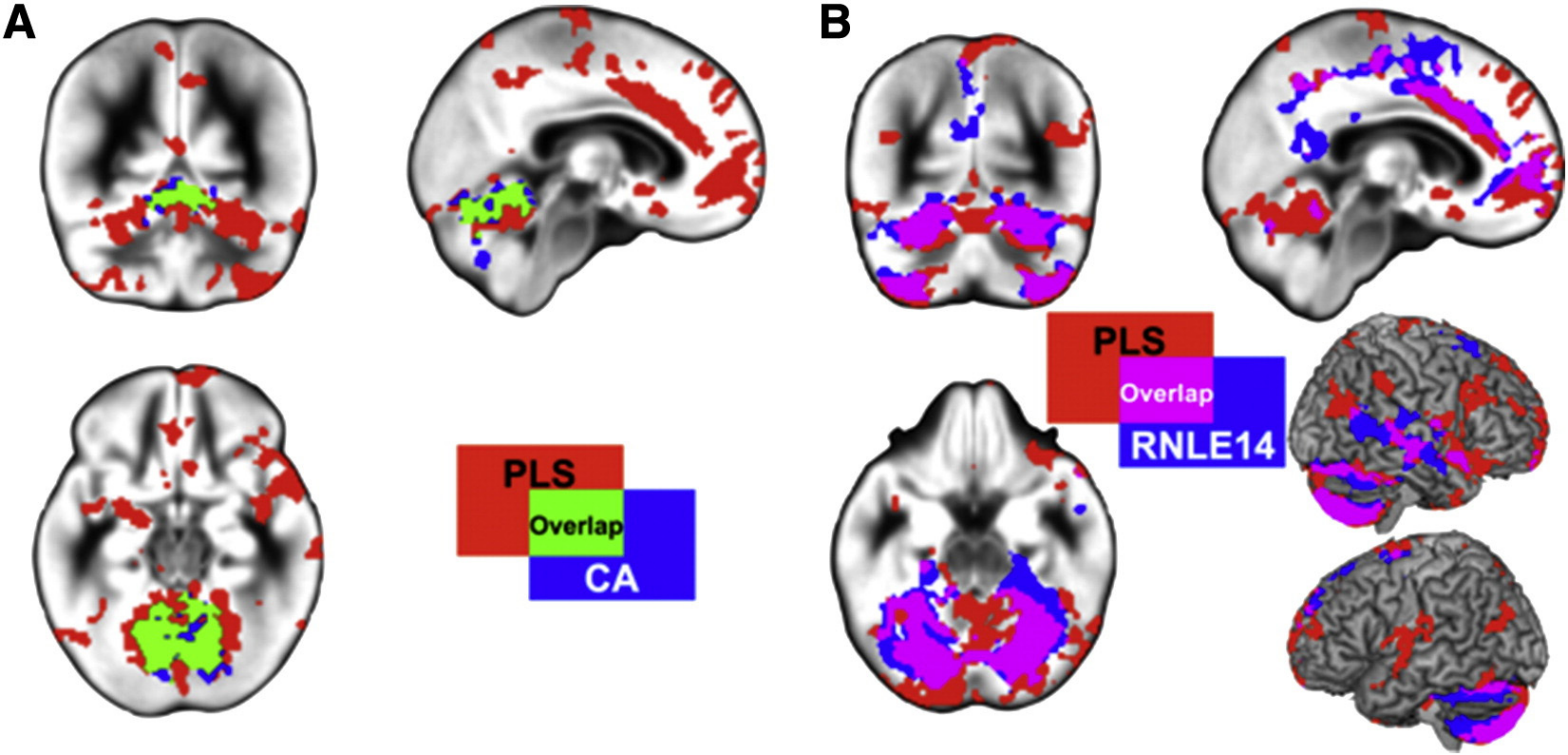
A) Conjunction analysis of multivariate and CA regions identified in univariate analyses. Red = regions identified in multivariate analysis; blue = CA regions in univariate analysis; green = overlap. B) Conjunction analysis of multivariate and RNLE14 regions identified in univariate analyses. Red = regions identified in multivariate analysis; blue = RNLE14 regions in univariate analysis; purple = overlap.

Using conjunction analysis we assessed whether the cerebellum cluster identified in the univariate CA analysis corresponded to the cerebellum cluster identified in the multivariate analysis. Overlap between the two analyses was observed in the vermis and medial cerebellum region (see Fig. 4A), providing confirmatory evidence that the cerebellum cluster identified in the PLS analysis was driven by CA. We then performed a conjunction analysis between RNLE14 and the multivariate results. Overlap was observed between the two analyses predominantly in the lateral cerebellum, cingulate/MPFC and right STS/STG (see Fig. 4B), providing confirmatory evidence that these regions in the PLS analysis were driven by RNLE14.

## 4 Discussion

In a representative community sample of currently healthy adolescents recruited from a larger longitudinal cohort study, we observed that correlated adverse psychosocial factors occurring from childhood through to mid adolescence are associated with GMV throughout distributed neural systems as measured in late adolescence. This result is consistent with past work demonstrating an association between adverse psychosocial experiences and reduced GMV in the cerebellum. However, as far as we know however, this is the first illustration of such multivariate associations and increases in brain GMV in humans following negative life events aged 14. Taken together the results are a striking illustration of the influence and embedding of the effects of the psychosocial environment on structural brain development.

Through subsequent univariate analyses we found that some variables are likely to exert a unique influence on particular brain structures. Therefore, this study not only advances our understanding of how the general psychosocial environment (composed of multiple adverse psychosocial variables occurring over time) affects structural development of large-scale neural systems, but also suggests specificity between anatomical regions and individual psychosocial factors.

### 4.1 Childhood adversity and the cerebellum

We found a clear association between CA and reduced GMV. We observed reduced GMV in the midline cerebellum in both the multivariate and univariate analyses with the conjunction analysis indicating the variation to be most apparent in the vermis and the midline cerebellum (see Fig. 3). The finding suggests that relatively chronic exposure to moderate childhood adversities may specifically reduce cerebellar GMV but as these are cross-sectional findings antecedent differences in cerebellar architecture prior to exposure cannot be ruled out.

The association is consistent with prior studies demonstrating smaller cerebellar vermis GMV in individuals exposed to severe maltreatment, adversity and neglect (Baldacara et al., 2011a; Bauer et al., 2009; Carrion et al., 2009; De Bellis and Kuchibhatla, 2006; Edmiston et al., 2011; Hanson et al., 2010; Kumari et al., 2013; Sheffield et al., 2013). The present study uniquely extends such findings by showing that this association occurs with moderate but relatively chronic parental discord. Parental discord is a common trans-diagnostic risk factor for many psychiatric disorders (Kessler et al., 2010) and smaller cerebellar vermal GMV has been repeatedly reported in ADHD (Bledsoe et al., 2009), affective disorder (Baldacara et al., 2011a; Shah et al., 1992), Autism (Courchesne et al., 1988), bipolar disorder (Baldacara et al., 2011b), conduct disorder (Fairchild et al., 2011) and schizophrenia (Ichimiya et al., 2001; Loeber et al., 2001). A smaller cerebellar vermis may therefore be a trans-diagnostic neural marker of psychopathology.

The cerebellum is differentially susceptible to the rearing environment and increased resting-blood flow in the cerebellum has been reported in individuals exposed to abuse (Anderson et al., 2002). Long-term motor skill training in animals and humans induces structural and functional changes in the cerebellum (Hutchinson et al., 2003; Kleim et al., 2007). From a psychosocial risk perspective, isolation reared rhesus monkeys demonstrated altered morphology of cerebellar Purkinje cells compared to colony reared primates (Floeter and Greenough, 1979) indicating negative effects of a depriving environment. The cerebellum vermis is also activated during states of high autonomic cardiovascular arousal such as exercise and mental stressor tasks (Critchley et al., 2000). Therefore a sustained activation following exposure to CA may be adaptive in the short-term but maladaptive over the long-term.

As the neonatal cerebellum contains the highest number of glucocorticoid receptors in the brain (Pavlik and Buresova, 1984; Sanchez et al., 2000) it may be particularly sensitive to allostatic failure in the presence of common family-focused CA (Wilkinson and Goodyer, 2011). However, we cannot rule out the possibility that smaller vermis GMV is due to negative in utero influences that may co-occur with CA or indeed as a result of as yet unidentified genetic variants. Currently, it is unclear what the functional implications of a smaller cerebellar vermis are in terms of emotion, motivational and cognitive processing. Infants with atypical neural development that involves congenital abnormalities of the cerebellar vermis show a range of behavioral and cognitive deficits (Pierce and Courchesne, 2001; Steinlin, 2008). Whether these apply, at a more subtle level to typically developing human infants who are exposed to a sub-optimal rearing environment is unclear.

### 4.2 Past psychiatric history and the medial temporal lobe

The finding of lower GMV in the right uncus/parahippocampal associated specifically with participant's lifetime psychiatric history suggests either 1) an effect whereby lower GMV in this region is a neuroendophenotype risk for subsequent mental illness or 2) a prior mental illness, most likely affective in nature, leads to reduced GMV, in line with the neural scarring hypothesis (Dannlowski et al., 2012; Wichers et al., 2010). Both are biologically plausible; and as we have shown, clearly not a consequence of any other correlated liability measured in this study. Decreased medial temporal lobe volume is a common finding in cross-sectional and prospective stress studies in healthy individuals (Gianaros et al., 2007; Papagni et al., 2011; Zannas et al., 2013) and those with psychiatric diagnoses such as Affective Disorders (Bora et al., 2012; Frodl et al., 2008), and Psychosis (Bodnar et al., 2012). Such a decrease may be an adaptive consequence of prolonged activity in this region during heightened states of anxiety (Osuch et al., 2000). Prior studies attributing smaller MTL volume to CA may have also failed to take subsequent episodes of mental illness into account (Moffitt et al., 2010; Rohde et al., 2013).

### 4.3 Recent negative life events and the adolescent brain

In contrast to the above findings, there was a significant and unexpected positive association of recent stressful life events recalled at age 14, upon brain GMV in cortical midline PFC and parietal areas, superior temporal areas and lateral cerebellum (see Fig. 2). Previously, such increases in these particular regions have been reported in prospective primate studies of monkeys exposed to prior stressors (Katz et al., 2009; Spinelli et al., 2009). The current findings in well individuals clearly require replication in an independent sample. One working hypothesis is that individuals exposed to this level of stressor at this earlier age may reflect a “stress-inoculation” or “steeling” resilience effect on neural development (Parker and Maestripieri, 2011; Parker et al., 2004; Rutter, 2012b). Significantly increased volume of the PFC has been reported in pediatric-PTSD samples compared to control children (Carrion et al., 2009; Richert et al., 2006). Additionally in the Richert et al. (2006) study those children with PTSD diagnoses and with the greatest reported functional impairment, demonstrated the greatest volume reduction in dorsal medial PFC, suggesting that it is the degree to which stressors functional impair an individual that affects brain GMV. We also found that there were no such effects for negative life events reported at age 17 which were more proximal to the scanning study. This was despite the frequency of reported events being higher at this age compared to those reported at age 14. Therefore, at present, we cannot give a definitive explanation of our opposing findings concerning CA and RNLE14 effects upon GMV. One reason may have to do with the point at which exposure occurred during development, with CA exposure earlier from birth to age 11, whereas RNLE14 occurred later than this. Relatedly it may be due to exposure occurring prior or after the onset of puberty in participants (CA occurring prior to and RNLE14 occurring during/after puberty). Another reason may be due to the severity of exposure, with RNLE14 exposure being relatively low (compared to RNLE17). Another reason may be due to the provider of information with CA information being reported by the parent whereas RNLE14 information being reported by the participant. However, due to the lack of effect due to RNLE17 exposure, this argues against this interpretation of the results. Future studies are required to clarify the effects these factors have upon GMV and further examine the role of recent negative life events upon brain GMV and especially type, duration, frequency and developmental timing of negative life events.

Future studies would need to take into account normative variation in developmental trajectories to make better sense of the impact of the proximal as well as the distal social environment. Previous studies have shown that the medial prefrontal cortex and posterior superior temporal sulcus are areas that show decreased thickness from 14 to 18 years of age (Mills et al., 2014), while the cerebellum shows an upward trajectory during this point in development (Tiemeier et al., 2010).

### 4.4 Adversities and the hippocampal and amygdala regions

Other notable regions of interest justified by past literature such as the hippocampus and amygdala were not associated with any one specific psychosocial variable we investigated but were identified in the multivariate analysis. This suggests that GMV measured in late adolescence in these key regions involved in emotion processing are either antecedent risks or sensitive to the chronic interplay of psychosocial adversities over time rather than vulnerable to a specific type of adversity.

### 4.5 5HTTLPR, adversities and lower GMV

Finally, in contrast to previous studies in humans and animals (Canli et al., 2006; Pezawas et al., 2005; Selvaraj et al., 2011), but in support of recent larger-sampled studies (Cole et al., 2011) we did not find significant effects of 5-HTTLPR variation on GMV, either as a main effect or in interaction with CA.

### 4.6 Limitations

The current findings are cross-sectional and prevent causal interpretations being made; thus whilst we demonstrate a multivariate set of associations with some demonstrable specificities, prospective studies are required to test our hypothesis of differential effects of psychosocial experiences on the developing brain. It is additionally important to exclude potential antecedent differences arising from latent genetics, congenital factors or other neutrally relevant toxins such as inflammation. A particular difficulty for this and many imaging studies is the absence of normative developmental brain map as a reference point for interpreting differences in case–control studies. It would therefore also be advantageous in future studies to have repeated neuroimaging scans to dynamically understand the moderating effects of environmental variables over the life course. Other limitations include the use of reliance of information obtained from maternal interview using the CAMEEI and the relatively high SES background of participants in this study that may contrast with the SES of participants in other studies who may have experienced more severe forms of abuse and adversity.

## 5 Conclusions

The findings show that moderate and chronic childhood adversities characterized by inter-parental discord are associated with widespread changes in GMV in the late adolescent brain. Further it was possible to reveal putative specific effects for some of these correlated psychosocial factors on regional changes in GMV. These findings suggest that a smaller cerebellar vermis may act as a trans-diagnostic marker for psychopathology. There may also be developmentally mediated effects of subsequent psychosocial risks on other later maturing brain areas such as reduced medial temporal lobe GMV associated with a psychiatric diagnosis and widespread increased GMV associated with negative life events aged 14. This study demonstrates that the developing brain may be sensitive to more common, moderate but chronic family-focused forms of adversities, as well as severe forms of maltreatment.

## Financial disclosures

Ian Goodyer has received payment from Janssen for lectures.

## Figures and Tables

**Table 1:** Characteristics of the ROOTS and neuroimaging samples classified by childhood adversity (CA) group.

Variable	Sample			
	ROOTS total		Neuroimaging	
	CA +	CA −	CA +	CA −
N =	292	776	27	31
Age at last assessment (Y/M) [Mean (SD)]	17.5 (0.3)	17.5 (0.3)	18.4 (0.6)	18.4 (0.7)
Gender (M/F)	139/153	334/442	10/17	16/15
⁎Socioeconomic status (ACORN measure) [N/%]				
Wealthy/urban	144 (49%)	529 (68%)	17 (63%)	18 (58%)
Comfortable	88 (30%)	164 (21%)	6 (22%)	10 (32%)
Moderate means/hard-pressed	60 (21%)	83 (11%)	3 (15%)	3 (10%)
5-HTTLPR genotype frequencies ss/sl/ll	54/134/92	134/394/225	11/0/16	14/0/17
IQ [Mean (SD)]	–	–	107 (9)	106 (10)
⁎MFQ [Mean (SD)]	16.7 (12.9)	13.4 (10.8)	11.7 (8.6)	8.5 (7.6)
⁎,#Participant psychiatric history [present/non-present %]	28/72	13/87	56/44	19/81
⁎,#Parental psychiatric history [present/not-present %]	69/31	34/66	30/70	35/65
⁎,#FAD [Mean (SD)]	24.3 (6.5)	21.9 (6.2)	25.4 (6.8)	21.5 (5.3)
⁎RNLE14 [Mean (SD)]	0.7 (1.1)	0.5 (0.9)	0.3 (0.6)	0.5 (0.8)
RNLE17 [Mean (SD)]	0.9 (1.2)	0.8 (1.2)	1.3 (1.7)	0.6 (0.7)

⁎ Significant difference at p < 0.05 in ROOTS sample.

# Significant difference at p < 0.05 in neuroimaging sample.

**Table 2:** Multivariate PLS results of relationships between adverse psychosocial variables on regional GMV.

Region	Label	Hemi	MNI x	MNI y	MNI z	Bootstrap Ratio	Cluster Size (voxels)
Limbic	vIns	L	− 45	− 3	− 18	4.8993	348
	sgACC/Nacc	B	7.5	18	− 16.5	3.9991	331
	Amyg/SI	L	− 27	0	− 16.5	3.9912	584
Cerebellum	Cerebellum	B	− 18	− 67.5	− 24	6.2607	20835
	Cerebellum	R	34.5	− 79.5	− 49.5	5.6175	3877
Temporal	STG/STS	L	− 52.5	− 7.5	− 3	4.8502	338
	MTG	R	58.5	− 31.5	1.5	4.7614	1115
	TP	R	19.5	9	− 45	4.761	405
	STG	L	− 66	− 3	16.5	4.6359	460
Lateral parietal/occipital	SMG/IPL	R	63	− 42	42	5.1726	340
	Ang/MOG	R	45	− 69	27	5.2282	1014
	MOG	L	− 48	− 72	18	4.5995	419
Midline parietal	Prec	R	3	− 58.5	46.5	4.0305	425
	Prec	R	6	− 63	64.5	4.5303	670
	RSC	B	1.5	− 54	12	4.2976	326
Midline prefrontal	ACC	B	1.5	21	31.5	5.805	7807
	SMA	R	9	− 18	78	5.1199	651
	dMPFC/ACC	B	− 7.5	36	40.5	5.336	870
	PMd/SMA	L	− 16.5	− 9	76.5	5.2981	1083
Lateral prefrontal	aPFC	L	− 24	55.5	15	4.3216	311
	aPFC	R	27	48	18	4.2848	258
	plOFC/ATL	R	33	27	− 24	5.7124	2876
	DLPFC/FO	R	45	13.5	30	5.0425	736

Brain regions: ACC = anterior cingulate cortex; plOFC = posterior lateral orbitofrontal cortex; ATL = anterior temporal lobe; dMPFC = dorsomedial prefrontal cortex; PMd = dorsal premotor cortex; SMA = supplementary motor area; Ang = angular gyrus; MOG = middle occipital gyrus; SMG = supramarginal gyrus; IPL = inferior parietal lobule; FO = frontal operculum; DLPFC = dorsolateral prefrontal cortex; vIns = ventral insula; STG = superior temporal gyrus; STS = superior temporal sulcus; MTG = middle temporal gyrus; Prec = precuneus; RSC = retrosplenial cortex; aRLPFC = anterior rostro-lateral prefrontal cortex; sgACC = subgenual ACC; NAcc = nucleus accumbens; Amyg = amygdala; SI = substantia innominata.

**Table 3:** Effects of childhood adversities (CA), participant psychiatric history, and recent negative life events aged 14 (RNLE14) on gray matter volume evident at the whole-brain level and surviving either FWE at p < 0.05 (#) or cluster-FDR correction for multiple comparisons at q < 0.05 (*). Effects evident after controlling for other confounding variables: gender, current depressive symptoms, 5-HTTLPR genotype variation, FAD questionnaire score, recent negative life events aged 17 and total intra-cranial volume. Abbreviations: SVC = Small Volume Corrected, FWE = Family Wise Error Corrected for multiple comparisons, FDR = False Discovery Rate.

Contrast	Region	Cluster size (ke)	T-score	Z-score	MNI X	MNI Y	MNI Z
CA − > CA +	Cerebellum⁎	5078	4.86	4.36	3	− 57	− 12
No psych history > psych history	Uncus#	1716	5.37	4.73	18	3	− 36
Positive effect of RNLE14	Mid. temp. gyrus.⁎	3676	6.06	5.20	60	− 30	− 5
	Cerebellum⁎	3183	5.11	4.54	33	− 49	− 50
	Cerebellum⁎	19573	4.99	4.45	30	− 55	− 29
	Cerebellum⁎	3486	4.83	4.34	− 32	− 63	− 51
	ACC⁎	3131	4.81	4.32	− 12	50	− 2

⁎ Significant at q < 0.05 FDR.

# Significant at p < 0.05 FWE.
