# Supplementary material for: General and specific effects of early-life psychosocial adversities on adolescent grey matter volume
Source: Neuroimage Clin. 2014 Jan 11;4:308–18. doi: 10.1016/j.nicl.2014.01.001 (PMC4107373; doi:10.1016/j.nicl.2014.01.001)
Supplement: Inline Supplementary Table S1 [file mmc1.docx]

Table S1: Neuroimaging sample participant psychiatric history as diagnosed using the K-SADS.

| **Group** | **Previous disorder** |
| --- | --- |
| CA+ | Previous NSSI, affective disorder(MDD), anxiety disorder (Panic disorder) |
| CA+ | Previous anxiety disorder (Specific phobia) |
| CA+ | Previous anxiety disorder (Specific phobia) |
| CA+ | Previous NSSI, affective disorder (MDD), Anxiety disorder (Anxiety NOS) |
| CA+ | Previous NSSI |
| CA+ | Previous behavioral disorder (CD, ODD, ADHD) |
| CA+ | Previous anxiety disorder (Panic disorder) |
| CA- | Previous affective disorder (MDD), anxiety disorder (Specific spider phobia) |
| CA- | Previous affective disorder (MDD), previous anxiety disorder (Panic attack) |
| CA- | Previous NSSI, previous MDD |
| CA- | Previous anxiety disorder (OCD & Panic attacks) |
| CA+ | Previous NSSI, affective disorder (MDD), anxiety disorder (Panic disorder), alcohol abuse |
| CA+ | Previous NSSI, affective disorder MDD, anxiety disorder (Panic disorder) |
| CA+ | Previous NSSI |
| CA+ | Previous eating disorder |
| CA+ | Previous affective disorder (MDD) |
| CA- | Previous behavioral disorder (ADHD) |
| CA- | Previous alcohol abuse |

**Abbreviations:**

NSSI (Non-Suicidal Self Injury)

MDD (Major Depressive Disorder)

NOS (Not Otherwise Specified)

CD (Conduct Disorder)

ODD (Oppositional Defiant Disorder)

OCD (Obsessive Compulsive Disorder)

ADHD (Attentional Deficit Hyperactivity Disorder)
