# Supplementary material for: General and specific effects of early-life psychosocial adversities on adolescent grey matter volume
Source: Neuroimage Clin. 2014 Jan 11;4:308–18. doi: 10.1016/j.nicl.2014.01.001 (PMC4107373; doi:10.1016/j.nicl.2014.01.001)
Supplement: Inline Supplementary Table S2 [file mmc2.docx]

Table S2: Neuroimaging sample - parental psychiatric history.

| **Group** | **Parental diagnosis** |
| --- | --- |
| CA+ | Mother Anxiety Disorder/MDD |
| CA+ | Mother MDD |
| CA+ | Mother MDD |
| CA- | Father MDD |
| CA- | Father MDD |
| CA+ | Mother MDD |
| CA- | Mother MDD |
| CA+ | Mother MDD, Father MDD |
| CA+ | Mother MDD, Father MDD |
| CA- | Father MDD |
| CA- | Mother MDD |
| CA- | Mother MDD |
| CA+ | Mother MDD |
| CA+ | Mother Dysthymia |
| CA+ | Mother MDD |
| CA+ | Father Alcohol/Substance Abuse |
| CA+ | Mother MDD, Father Alcohol/Substance Abuse |
| CA+ | Mother Eating Disorder, MDD |
| CA+ | Mother MDD |
| CA+ | Father MDD |
| CA+ | Mother MDD, Father Alcohol/Substance Abuse |
| CA- | Mother Anxiety Disorder |
| CA- | Father MDD |
| CA- | Mother MDD, Father Bipolar Disorder |
| CA+ | Mother MDD, Personality Disorder |
| CA- | Mother MDD |
| CA- | Mother MDD |
| CA+ | Mother MDD |
| CA+ | Mother MDD and Dysthymia |
| CA+ | Mother MDD |
| CA+ | Mother MDD |
| CA+ | Mother Dysthymia, Father Alcohol/Substance Abuse |
| CA+ | Mother MDD |

**Abbreviations:**

MDD (Major Depressive Disorder)
